# Y-chromosome and Surname Analyses for Reconstructing Past Population Structures: The Sardinian Population as a Test Case

**DOI:** 10.3390/ijms20225763

**Published:** 2019-11-16

**Authors:** Viola Grugni, Alessandro Raveane, Giulia Colombo, Carmen Nici, Francesca Crobu, Linda Ongaro, Vincenza Battaglia, Daria Sanna, Nadia Al-Zahery, Ornella Fiorani, Antonella Lisa, Luca Ferretti, Alessandro Achilli, Anna Olivieri, Paolo Francalacci, Alberto Piazza, Antonio Torroni, Ornella Semino

**Affiliations:** 1 Dipartimento di Biologia e Biotecnologie “L. Spallanzani”, Università di Pavia, 27100 Pavia, Italy; viola.grugni@unipv.it (V.G.); alessandro.raveane01@universitadipavia.it (A.R.); giulia.colombo01@universitadipavia.it (G.C.); nici.carmen@gmail.com (C.N.); f.crobu@irgb.cnr.it (F.C.); linda.ongaro@ut.ee (L.O.); vincenza.battaglia@unipv.it (V.B.); darsanna@uniss.it (D.S.); nadia.alzahery@unipv.it (N.A.-Z.); luca.ferretti@unipv.it (L.F.); alessandro.achilli@unipv.it (A.A.); anna.olivieri@unipv.it (A.O.); antonio.torroni@unipv.it (A.T.); 2 Istituto di Ricerca Genetica e Biomedica, Consiglio Nazionale delle Ricerche (CNR), 09042 Monserrato, Italy; 3 Estonian Biocentre, Institute of Genomics, Riia 23, 51010 Tartu, Estonia; 4 Department of Evolutionary Biology, Institute of Molecular and Cell Biology, Riia 23, 51010 Tartu, Estonia; 5 Dipartimento di Scienze Biomediche, Università di Sassari, 07100 Sassari, Italy; 6 Istituto di Genetica Molecolare “L.L. Cavalli-Sforza”, Consiglio Nazionale delle Ricerche (CNR), 27100 Pavia, Italy; fiorani@igm.cnr.it (O.F.); antonella.lisa@igm.cnr.it (A.L.); 7 Dipartimento di Scienza della Vita e dell’Ambiente, Università di Cagliari, 09123 Cagliari, Italy; paolo.francalacci@unica.it; 8 Dipartimento di Scienze Mediche, Scuola di Medicina, Università di Torino, 10124 Torino, Italy; alberto.piazza@unito.it

**Keywords:** Human Y-chromosome variation, haplogroups, family name origin, migrations, peopling of Sardinia, phylogenetics

## Abstract

Many anthropological, linguistic, genetic and genomic analyses have been carried out to evaluate the potential impact that evolutionary forces had in shaping the present-day Sardinian gene pool, the main outlier in the genetic landscape of Europe. However, due to the homogenizing effect of internal movements, which have intensified over the past fifty years, only partial information has been obtained about the main demographic events. To overcome this limitation, we analyzed the male-specific region of the Y chromosome in three population samples obtained by reallocating a large number of Sardinian subjects to the place of origin of their monophyletic surnames, which are paternally transmitted through generations in most of the populations, much like the Y chromosome. Three Y-chromosome founding lineages, G2-L91, I2-M26 and R1b-V88, were identified as strongly contributing to the definition of the outlying position of Sardinians in the European genetic context and marking a significant differentiation within the island. The present distribution of these lineages does not always mirror that detected in ancient DNAs. Our results show that the analysis of the Y-chromosome gene pool coupled with a sampling method based on the origin of the family name, is an efficient approach to unravelling past heterogeneity, often hidden by recent movements, in the gene pool of modern populations. Furthermore, the reconstruction and comparison of past genetic isolates represent a starting point to better assess the genetic information deriving from the increasing number of available ancient DNA samples.

## 1. Introduction

Sardinians, albeit clearly Europeans, represent the main outlying gene pool in the European genetic landscape [1,2,3,4,5]. To understand the origin and the evolutionary forces at the basis of their differentiation, Sardinians have been the subject of numerous genetic, linguistic and anthropological analyses. According to linguistic studies, the origin of Sardinians pre-dates the settlement of the Indo-Europeans in western Europe [6,7,8]. The earliest archaeological evidence of modern humans in the island goes back to the Paleolithic (between 20 to 14 kya), when Sardinia and Corsica were a single land, yet separated from the mainland [9,10,11,12]. Initially, the island population was small; it gradually increased in the Neolithic period and later, especially in the Bronze Age with the development of the advanced civilization characterized by the *nuraghi*, megalithic edifices of a circular shape very similar to buildings observed in other islands of the Mediterranean Basin. Afterwards, the population size remained approximately constant until the last three centuries when it underwent a significant growth [11]. Following the first settlement, the most important external contributions were provided by the Phoenicians (9th century BCE) and the Carthaginians (5th century BCE), who controlled the entire island, with the only exception of the region of Olbia, ruled by the Greeks [11]. At the end of the First Punic war (238 BCE), Sardinia passed under the control of Rome. Yet, many archaeological remains prove that the influence of the Romans and previous conquerors was limited to the coastal regions, whereas the mountainous central district of the island, the so-called “archaic zone”, became the refuge of the indigenous non-Indo-European inhabitants of Sardinia (*Nuragians*). Likewise, subsequent invasions by Vandals (456 CE), Byzantines (534 CE), Saracens (7–10 century CE) and Pisans (1052–1295) had a limited impact [13]. With regards to the Spanish, who ruled the island until 1713, they did have an important cultural impact in the North-West of the island, as attested by the language spoken there [14]. Eventually, from 1718 to 1820 Sardinia was annexed by the Savoy.

In spite of this continuous chain of invasions, the gene flow into the Sardinian population has been relevant only in some coastal areas where well-known foreign settlements took place [15]. As a result, the ancient origin of native Sardinians and their long-standing isolation might provide an explanation for their genetic peculiarity [16], characterized by high frequencies of uniparental haplotypes that are rare elsewhere in Europe [2,17,18,19,20,21,22,23], extensive linkage disequilibrium of autosomal markers [24], as well as high degrees of homozygosity at the genomic level [25].

However, a main settlement in Neolithic times followed by long-lasting isolation can also explain the extreme similarity at the nuclear genomic level with early European Neolithic farmers [26,27] and with the Late Neolithic/Chalcolithic Tyrolean Iceman [28,29], but not the similarity observed with the Near Eastern Neolithic farmers including those from Anatolia [30]. The small population size (from pre-history to 1700 CE the Sardinian population never exceeded 300 thousand inhabitants, and in around 1348 CE, the Black Plague reduced the population by half), the presence in the island of natural barriers such as mountains, but also the endemic malaria in the lower lands, which kept certain areas very isolated [31], contributed to creating different genetic isolates and consequently heterogeneity within regions. On the whole, three large areas of Sardinia reflecting its ancient history and geography were identified. The northern zone is delimited by the mountain chain crossing Sardinia from the Central-West to the North-East and is linguistically different from the rest of the island. The south-western zone is delineated by the presence of many Phoenician and Carthaginian archeological sites [13]. The central-eastern zone is the asylum land of the ancient Sardinian population during invasions and is a domain of pastoral culture. This zone includes the more conservative or “archaic” area, defined by archaeological, linguistic [32], geo-linguistic and genetic factors [8] (for a more detailed subdivision of Sardinia on the basis of genes, languages and surnames, see [14]).

Although genetic differences are still detectable between communities [33,34,35,36,37,38,39,40,41,42], the increasing internal migration toward the main villages and towns of the last 150 years has weakened and sometimes erased the boundaries of these isolates, partially blurring the ancient genetic structure of the island [2,4,20] and making it difficult to reconstruct its past demographic history. However, the results of a previous study indicate that the use of a sampling method based on the geographic origin of family names (territorial monophyletic family names), in comparison with the usual grandparent’s birthplace sample collection strategy, allows, for the Y-specific gene pool at least, the reconstruction of ancient isolates, bypassing the effect of recent migrations [19].

Thus, this study exploited a sampling strategy based on the origin of the family name [19] and a detailed Sardinian Y-chromosome phylogeny [22,43] to reconstruct ancient genetic isolates of the Sardinian male component and to address the following questions: can we detect the ancient heterogeneity in the actual Sardinian gene pool? And, if so, what information does it provide about the early peopling of the island and subsequent migrations?

To answer these questions, Y-chromosome high-resolution analyses were performed on 603 Sardinian males representative of the different zones of the island, after having also assessed the linguistic and geographic origins of their family names. Our results provide new clues for understanding the fine genetic structure of the Sardinian population, an essential piece of information not only in an evolutionary context, but also for reducing confounding effects caused by population structure in association studies.

## 2. Results

### 2.1. Classification and Distribution of Y-chromosome Haplogroups in Ancient Isolates of Sardinia

**Figure 1:**
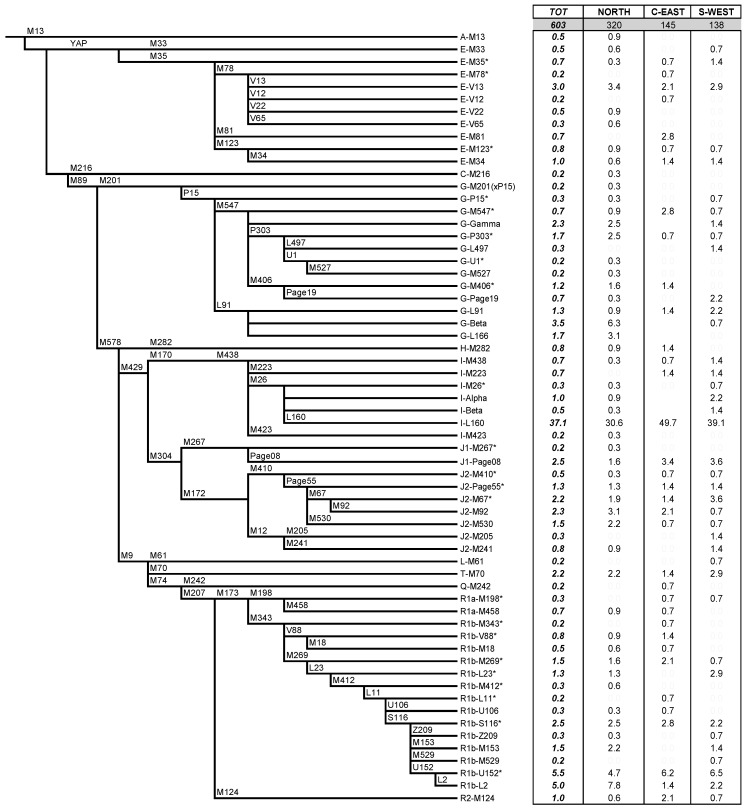
Phylogenetic tree of Y-chromosome haplogroups and their frequencies (as percentage) in the whole Sardinian sample and in the three main geo-cultural regions of the island. Names of markers are indicated above the lines; the lengths of branches are not drawn to scale for better readability. Asterisk (*) indicates a paragroup: group of Y chromosomes defined by the derived state of the main haplogroup but by any downstream mutation.

**Figure 2:**
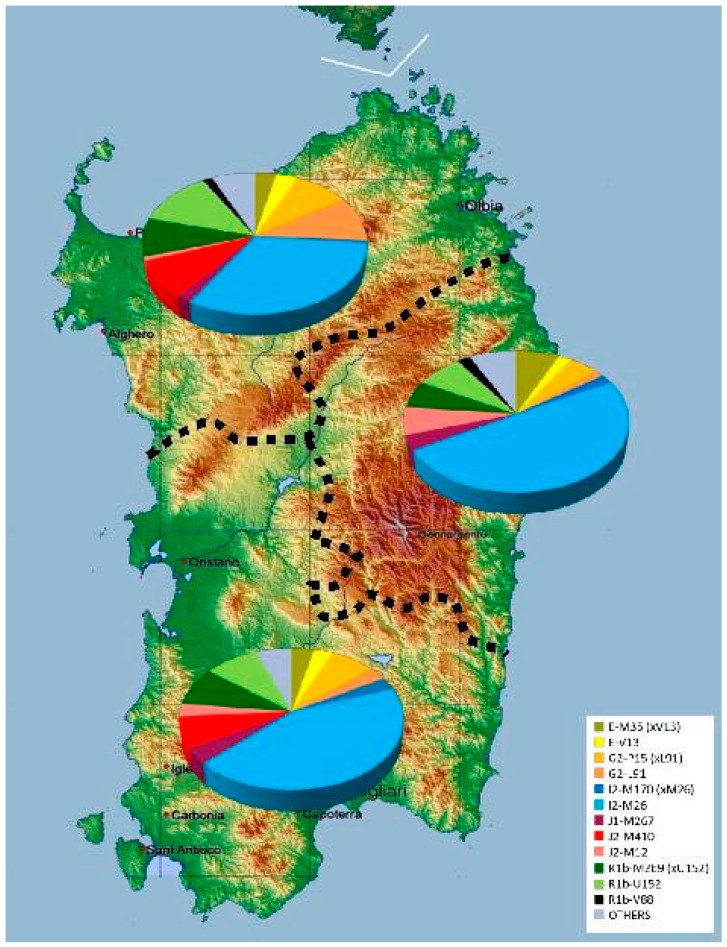
Proportion of the observed Y-chromosome haplogroups in the three main cultural and geographical areas of Sardinia. Dashed lines indicate boundaries of the three main areas.

The analysis of 603 subjects with monophyletic surnames allowed the identification of 62 Y-chromosome lineages belonging to 14 main Y-chromosome haplogroups (Hgs). The relative frequencies of the haplogroups observed in the global sample and in the three main areas of Sardinia are listed per haplogroup in the table of Figure 1 and are summarized in Figure 2.

No significant difference in the haplogroup profiles was observed in comparison with previous datasets [18,19,20,44,45,46], thus showing that our “monophyletic” sample well represents Sardinian variability.

The most frequent haplogroups are I-M170 (41.7%) (almost exclusively represented by its sub-clade I2-M26 (38.9%)) and R1-M207 (21.1%) (represented mainly by its branch R1b-M269: 18.6%), followed by G-M201 (14.3%) (with its most frequent sub-haplogroup G2-L91: 6.5%) and J-M304 (11.06%) (with its most frequent sub-haplogroup J2-M410: 7.8%). Haplogroups observed in southern Europe such as the Balkan E-V13 (3.0%), the Arab J1-M267 (2.7%), the African E-M33, E-M81, E-V12, E-V22, E-V65 (2.2%) and peculiar haplogroups such as A-M13 (0.5%) common in East Africa, R1b-V88* (0.8%) and its derivative R1b-M18 (0.5%), as well as R2-M124 (1.0%) observed mainly in South West Asia, were also detected. In addition, the rare lineage H2-M282 characterizes 0.8% of our Sardinian sample.

After the re-distribution of the subjects in the different regions of the island according to the origin of their family name (Figure S1), a general heterogeneity in the allocation of many haplogroups emerged (χ^2^_[df112]_ = 164.96; *p* < 0.001) with only 21 out of the 62 defined lineages shared among the three main geographic regions (Table of Figure 1). In particular, the χ2 per cell analysis (Table S1) showed significantly higher than expected frequencies of haplogroups G2-L91 and R1b-L2 in the northern areas compared to frequencies in the central region, which, in turn, is characterized by a significantly higher incidence (50%) of the lineage I2-M26, represented almost exclusively by its sub-clade I2-L160.

### 2.2. Sardinian Populations in the Mediterranean Context

**Figure 3:**
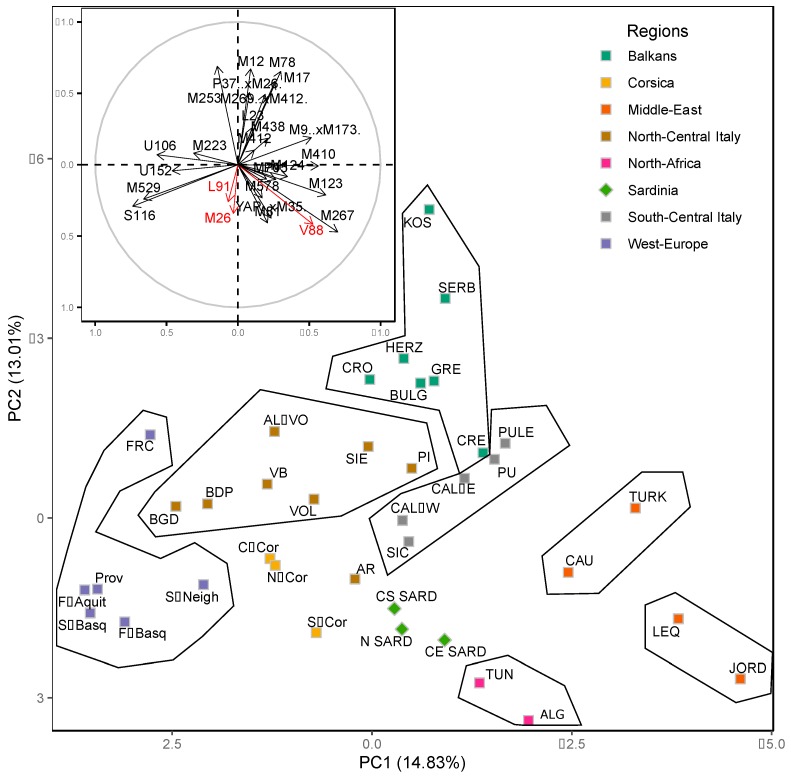
Principal component analysis performed using haplogroup frequencies in the Sardinian samples of the present study compared with those of relevant populations from the literature at the highest possible level of resolution (Table S2). Of the total variance, 27.84% is represented: 14.83% by the first PC and 13.01% by the second PC. The inset illustrates the contribution of each haplogroup. S-Neigh: Spanish from Western Bizkaia, Cantabria, Burgos, La Rioja, North Aragon; S-Basq: Spanish Basques from Roncal, Naffarroa, Gipuzcoa, Araba, Bizkaia; F-Basq: French Basques from Soule, Aquitani; F-Aquit: Franch from Bigorre, Bearn, Chalosse; FRC: France; PROV: Provence; N-COR: Corsica-North; C-COR: Corsica-Central; S-COR: Corsica-South; N SARD: Sardinia-North; CE SARD: Sardinia-Central East; CS SARD: Sardinia-Central South; AL-VO: Alessandria-Voghera, Piedmont; VB: Borbera Valley, Piedmont; BDP: Bergamo Plain, Lombardy; BGD: Bergamo Valley, Lombardy; VOL: Volterra, Tuscany; PI: Pisa, Tuscany; AR: Arezzo, Tuscany; SIE: Siena, Tuscany; PU: Apulia; PULE: Apulia-Grecanica; CAL-E: Calabria Ionian; CAL-W: Calabria Tyrrhenian; SIC: Sicily; CRO: Croatia Mainland; HERZ: Herzegovina; SERB: Serbia; BULG: Bulgaria; GRE: Greece Mainland; CRE: Crete; TURK: Turkey; CAU: Caucasus; LEQ: Lebanon+Iraq; TUN: Tunisia; ALG: Algeria.

In order to evaluate the position of Sardinians in a wider European and Mediterranean population context and visualize the relationships between Sardinians and other Italian populations, a Principal Component (PC) analysis was carried out on haplogroup frequencies, exploiting available literature data normalized to the highest possible level of phylogenetic resolution (Table S2). The plot of the two PCs is shown in Figure 3 together with a plot displaying the contribution of each haplogroup to the first and second PC.

The distribution, which is based on 27.84% of the total variance, shows an overall general agreement with geography: while the first component separates the populations according to longitude, the second component discriminates the populations approximately according to latitude. The continental populations from lands lying around the Mediterranean Sea are clearly separated at the periphery of the plot in five clusters (North Africa, Spanish, French and Basque populations, Balkans, Caucasus-Anatolia and Middle East). The populations of the Italian Peninsula and the Mediterranean islands follow a North-South cline with the northern-central Italians (together with Corsicans) closer to Spanish and French groups while the southern central Italians (and Sicilians) are closer to Caucasus-Anatolian and middle eastern populations. A similar pattern is also detected when looking at the entire genome, with Corsicans closer to the Central-North Italian groups [25], and Sicilians to Central-South Italian populations [5]. Conversely Sardinians, who strongly behave as outliers at the genomic level [5], appear located at a fringe of the Western European distribution of Y-chromosome variation. Such a position is explained by a markedly higher incidence of haplogroups I2-M26 and G2-L91 and by the presence of the R1b-V88 clade, virtually absent in other European populations [47].

### 2.3. The Founder Paternal Lineages G2-L91, I2-M26 and R1b-V88

Three haplogroups were previously identified as founding lineages: G2-L91, I2-M26 and R1b-V88.

Haplogroup G2-L91 (Table S3) reaches its highest frequency in southern Corsica (22.1%) and North Sardinia (10.5%). It appears at low frequencies elsewhere without any apparent pattern: in Iberia (0.3–1.0%), South France (0.8%), Continental Italy (1–1.2%), Austria (0.4%), Germany (0.3%), Czech Republic (2.9%), Armenia (0.9%), Iran (1.0%), Israel (0.3–0.6%), Egypt (4.1%) and in Moroccan Berbers (0.8%) [48,49]. This haplogroup characterizes also Ötzi, the Tyrolean Copper Age man [28,49].

The Network analysis of G2-L91 haplotypes (Table S4; Figure S2) reveals a composite pattern of evolution characterized by haplotypes widely shared between Corsican and Sardinian samples, suggesting a major demographic expansion in Corsica and by complex reticulations connecting the most extreme outlier haplotypes from the middle eastern, Egyptian but also Tyrolean samples. On the other hand, the Multi-Dimensional Scaling (MDS) analysis of the Short Tandem Repeat (STR) haplotype length variation (Figure S3) shows that Sardinian (and Corsican) G2-L91 chromosomes are much closer to the middle eastern chromosomes than to the European ones. Although the available data do not provide any indications about the dispersal route of G2-L91 in Europe and Africa, nor concerning its arrival in Corsica and Sardinia, it is interesting to note that the diffusion and microsatellite dating (Table S5) of this lineage along the coasts of the Mediterranean overlap that of Cardial Ware pottery [50]. This suggests at least two different dispersal routes of G2-L91 from the Middle East, where the haplotype diversity is the highest (Table S5): one through the Balkans up over and beyond the Alps, which would explain the presence of ancient and modern Tyrolean G2-L91 Y chromosomes; the second, likely by sea, which would explain the dispersal along the coasts of North African and southern Italy and the Mediterranean islands.

Although the highest frequency of G2-L91 is observed in Corsica, the highest STR variation of the lineage is detected in the northern area of Sardinia (Table S5), suggesting that the spread was from Sardinia to Corsica and not vice versa, as previously proposed [22,28,48]. On the other hand, the high frequency of G2-L91 in southern Corsica (Table S3) is characterized by a low diversity (Table S5), which could be explained by the flourish of the Torrean civilization, a Nuragic culture that spread to southern Corsica from North Sardinia in the Bronze Age [11].

Haplogroup I2-M26, likely of south-western European origin [51], accounts for more than one third of Sardinian Y-chromosomes, while it is rare in most other modern European populations, including the neighboring Corsicans (Table S6). Frequencies above 5% are observed only in Basque groups.

The dissection of this haplogroup into its main subclades (I2-M26alfa-Z27361, I2-M26beta-Z27401, I2-L160 and its sub-lineages) previously identified in Sardinia [43] highlights that the majority of the I2-M26 chromosomes belong to I2-L160, both in Sardinia and outside the island (Table S6, [52]). Three Volterra samples, two I2-M26*(xM26alpha-Z27361, M26beta-Z27401) and one I2-M26alpha-Z27361, and a Basque subject, I2-M26beta-Z27401 [22], are the only exceptions.

Network analyses reveal a massive expansion of sub-clade I2-L160delta in Sardinia (Figure S4; Table S7) and a local expansion in the British Isles of branches I2-M26(xL160) (Figure S5; Table S8) that do not involve haplotypes observed in Sardinia and south-western Europe. In both networks, Sardinian samples share haplotypes with, or are directly connected to, French/Spanish/Italian subjects. Interestingly, one of the two 5 ky old males buried in a Neolithic French necropolis and classified as I-P37 [53] shares its STR haplotype with Y chromosomes I2-M26(xL160) of modern French, Irish and Norwegian samples and I2-L160 of modern Spaniards, Sardinians and continental Italians (Table S8). Although the lack of a sub-classification of this ancient specimen and the low frequency of haplogroup I2-M26 outside Sardinia do not allow any inference about the source area of the Sardinian I2-M26 Y chromosomes, MDS analysis of the haplotype STR length variation highlights a closer relation between Sardinian and French samples (Figure S6). Based on microsatellite variability, [51] south-western Europe (Iberian Peninsula/southern France) is a likely area of origin for this haplogroup and a starting point for the first colonization of Sardinia. However, the observation of chromosomes I2-M26 (xM26alfa, M26beta) and I2-M26alfa (I-Z27361) in Tuscany highlights a possible alternative route for the arrival of haplogroup I2-M26 in Sardinia: from Tuscany through the islands of Elba and Corsica.

The sub-classification of the I2-L160 chromosomes into the main subgroups [I2-L160gamma (Z27138), I2-L160delta A (Y20194), I2-L160delta B (Z26452), I2-L160delta C (Z26534), I2-L160delta D (PF4225), I2-L160delta E (PF4265), I2-L160delta F (PF4301), I2-L160delta G (Z26723), I2-L160delta H (PF4364), I2-L160delta I (Z26773) and I2-L160delta L (PF4421)] (Table S9, Figure S4) revealed that some of them have comparable frequencies in the three areas of the island, while others are more frequent or unique to only one area (I2-L160delta G observed only in the South). Although the high level of resolution achieved in the sub-classification has strongly reduced the sample size of the identified sub-clades, preventing a statistically significant distribution being obtained of I2-L160 sub-lineages in the Sardinian isolates (X_26df_ = 28.4, *p*-value = 0.3), higher frequencies of I2-L160delta D (PF4225) in North Sardinia and I2-L160delta I (Z26773) in the central-eastern area of the island are apparent. It is of note that with the only exceptions of a I2-L160delta L (PF4421) Y chromosome in Calabria and a I2-L160delta A (Y20194) Y chromosome in Andalusia (easily explained as a recent Sardinian contribution), the I2-L160 sub-lines are virtually only observed in the island, supporting a Sardinian origin of I2-L160delta clades. This interpretation seems to be confirmed by the results obtained by the YFull Tree results [54].

Haplogroup R1b-V88 is a scarcely represented early branch of haplogroup R1b, mainly observed in sub-Saharan Africa. Its highest frequency is reported in Central Sahel (northern Cameroon, northern Nigeria, Chad and Sudan) where R1b-V88 sub-lineages underwent an expansion in Chadic-speaking groups [47,55,56]. Outside Africa, R1b-V88 lineages have been sporadically observed in the Middle East [57] and Europe, particularly in Sardinia [17,20,22,47,56,58]. Different hypotheses have been proposed concerning its ancestral homeland: a western Asian/middle eastern origin [47,59] and a sub-Saharan African origin [55] were hypothesized to explain its diffusion and variation in Africa, while no explanation was advanced for the presence of its sub-lineage R1b-M18 in Sardinia. The recent and detailed reconstruction of the phylogeny of this haplogroup [56] has revealed that the rare European R1b-V88 lineages (R1b-M18 and R1b-V35) originated from the root of the phylogeny much earlier (about 12.34 kya) than the separation of the African lineages (7.85 ± 0.90 kya), thus supporting an origin of R1b-V88 outside Africa and a subsequent diffusion in sub-Saharan Africa through the Last Green Sahara period during the Middle-Holocene [56]. Interestingly, recent studies on ancient DNA [60,61,62] identified the most ancient R1b-V88 samples (dated 11 and 9 ky) in East Europe (Serbia and Ukraine, respectively) and more recent R1b-V88 samples (dated 7 and 6 ky) in Spain (I0410) and Germany (I1593, I0559) thus supporting a European origin and opening new grounds for discussion concerning the routes towards Africa and Sardinia, where R1b-V88 characterizes a considerable number of ancient specimens [62,63].

### 2.4. Haplogroup Distribution in Pre-Historic Sardinian Samples

Ancient genomes of a number of Sardinian specimens, 44 of which males, derived from various caves located in different areas of the island and belonging to different archaeological phases, have been recently analyzed [62,63]. On the whole, nine Y-chromosome haplogroups have been identified, all of them observed in modern samples (Figure 1): E-L618 (derivative of E-M78, precursor of E-V13), G2-L166 (derivative of G-L91), G2-F872 (derivative of G2-L30, equivalent to G2-M547), I2-M223, I2-M26, I-M423, J1-L862 (derivative of J1-Page08), J2-M241 or J2-L283 (derivative of J2-M241), R1b-V88 and R1b-M269. In addition, chromosomes I2-M436(xM223) and R1b-L754(xM269) were also reported.

G2-L91, G2-F872 characterized the oldest specimens (from Middle to Late Neolithic), R1b-V88 and I2-M223 were observed in samples from the Early Copper Age and especially from the Early Bronze Age and the Nuragic period. I2-M26 appeared starting from the Early Bronze Age, J2-L283 from the Nuragic period and J1-L862 and R1b-M269 from the Punic period. Finally, haplogroups E-L618 and I2-M423 appeared only in Punic and Medieval specimens (Figure S7).

Haplogroup R1b-V88, observed in pre-Neolithic times in Balkan subjects [64], is the most represented haplogroup characterizing central-eastern and south-western Sardinian samples from the Early Copper Age (one subject in the South West), to the Nuragic period (four Early Bronze Age subjects all located in the central-eastern area; five subjects of Nuragic period-three in the central-eastern area and two in the south-western area). Haplogroup I2, which has been observed in pre-Neolithic times in Europe, is considered a hunter-gatherer signature. Together with haplogroup G2, it was common in Copper Age Iberia and appeared in Sardinia in Early Bronze Age as I2-M223, mainly in the North, and as I2-M26 in the Central-East area. Conversely, I2-M223 is observed in modern samples at low frequency (1.4%) only in the Central-East and South-West areas, while I2-M26 is present at high frequency on the entire island, especially in the Central-East area (49.7%). Haplogroups G2-F872 (M547) and J2-M241 emerge in the ancient genetic Sardinian landscape only in the Nuragic period. Haplogroup G2-F872 (M547), which characterizes three ancient DNAs (aDNAs) [62,63], one from Central-East, and two from South-West Sardinia and 7.3% of modern samples (with higher frequency in the North (8.4%) and lower in the South-West (6.4%) and Central-East (4.9%)), was described in an Anatolian sample older than 7 ky [65]. G2-L166 (L91) was observed in four aDNAs from northern Sardinia and in 6.5% of present-day Sardinians at a higher frequency in the North (10.3%) than in the Central-East and South-West areas. As previously mentioned, this haplogroup characterizes the 5.3 ky old Tyrolean Iceman while its precursor (G2a2a1) was commonly found in Anatolia and eastern European Neolithic specimens as well as in Chalcolithic Iberians [60,61,66,67]. Haplogroup R1b is represented in ancient samples mainly by derivatives of R1b-V88. This haplogroup, which likely originated in eastern Europe, where the most ancient samples (dated 11–9 ky) have been reported [61,62], characterizes Sardinian samples older than 5 ky. Taking into account that the European branches of R1b-V88 are different and phylogenetically older than the African ones [56], it is likely that R1b-V88 chromosomes reached Sardinia through western Europe. Interestingly, ancient R1b chromosomes have been described in Italy (Villabruna, dated 14 ky, [68]) and Iberia (dated 7 ky, [27]). On the other hand, haplogroup R1b-M269, common in the Iberian Peninsula since 4.5 kya, where it almost completely replaced the pre-existing haplogroups I2, G2 and R1b(xM269) [67] and is frequent (21.3%) in modern samples from North Sardinia, was observed in ancient DNAs from Punic and Medieval sites. The observation in Punic sites (South-West area) of haplogroups J1-L862 and J2-L283, described within Levantine Bronze Age individuals [69] and very common in modern North African [70] and Balkan populations [71], respectively, may represent traces of migrations from Levant/North African following the conquest of the island by the growing Carthaginians. Accordingly, these haplogroups in modern samples show their highest frequencies (3.6% and 1.4%, respectively) in the south-western area. Finally, haplogroup E-L618, described in a 15,000-year-old modern human from eastern Morocco attributed to the Iberomaurusian culture [72], may testify a further link with North Africa, although modern samples belonging to the equivalent haplogroup E-M78*(xV13) have been described in Egypt [73] and in the Balkans [71].

## 3. Discussion

Genome-wide analyses of modern DNA place Sardinian samples in an outlier position in the European genomic landscape [2,5,25,74,75,76] strongly mirroring the outcome of genomic analyses of early European Neolithic farmers [26,27]. The peculiarity of modern Sardinians is confirmed by the analysis of Y-chromosome haplogroup frequencies. Genetic drift and long isolation can explain the presence of haplogroups that are very rare in other European populations such as R1b-V88 and G2-L91 as well as the increase of frequency of haplogroup I2-M26. Indeed, as highlighted by the Principal Component Analysis plot (PCA, Figure 3), Sardinian groups are located at the boundary of the European distribution towards North Africa with a relative closeness to Corsican samples as also detected by genome-wide based haplotypes [5]. While the proximity with Corsican groups, especially of southern Corsicans and northern Sardinians, is due to the high incidence of haplogroup G2-L91 in both populations, the closeness to North Africa, mainly due to the sharing of R1b-V88, is overestimated since Sardinian and African R1b-V88 Y chromosomes belong to different sub-lineages that phylogenetically diverged more than 7 kya [56].

Although archaeological data indicate that Sardinia has been inhabited since Paleolithic times, this early human presence might have been very limited, and the time of the first peopling is still a matter of debate. Modern and ancient mitogenome analyses [23] have shown that the majority of Sardinian-specific mtDNA haplogroups coalesce in post-Nuragic, Nuragic and Neolithic-Copper Age periods, although some rare maternal lineages (K1a2d and U5b1i1) might have been on the island already in pre-Neolithic times. The recent analysis of ancient Sardinians belonging to different archaeological periods revealed a high level of genetic continuity with the Nuragic period for the male counterpart, also. Three Y-chromosome haplogroups (G2-L91, I2-M26, R1b-V88), which on the basis of ancient and modern samples had been previously proposed as founder lineages [22,28], were indeed all observed [62,63].

Although the demographic and genetic features associated with insularity offer the opportunity to better evaluate the impact of evolutionary forces such as founder events, gene flow and genetic drift that acted on the present Sardinian population, the homogenizing effect caused by internal movements during the last 150 years is a major confounding element. Here, through the “monophyletic surname sampling” approach, we obtained, at least for the Y-chromosome variation, novel clues concerning the past genetic isolates of the island, thus bypassing the confounding effect of recent migrations. With this approach we obtained a picture of the island population at the time of the introduction of the use of surnames, an event that in Italy occurred in the Middle Age. The comparison of the Y-chromosome haplogroups of ancient samples dated from the Early Neolithic [62] with those present in modern samples revealed a markedly different haplogroup distribution in the three large areas reflecting the ancient historic and geographic subdivision of the island (Figure S7). For some haplogroups the differences are likely the legacy of the ancient distribution showing local continuity albeit with varying frequencies. Thus, for example, G2-L91 shows its highest frequency (10.3%) in the North where it was also observed in aDNAs (four subjects), while it shows low frequencies in the Central-East (1.4%) and South-West (2.9%); I2-M26 characterizing two aDNAs, one from central-eastern and one from south-western areas, is found across the entire island at high frequency but especially in the Central-East. Differently, R1b-V88 is not observed in the South where it was observed in aDNAs; in contrast, I2-M223 was observed in different aDNAs in the North where it was not detected in modern samples.

**Figure 4:**
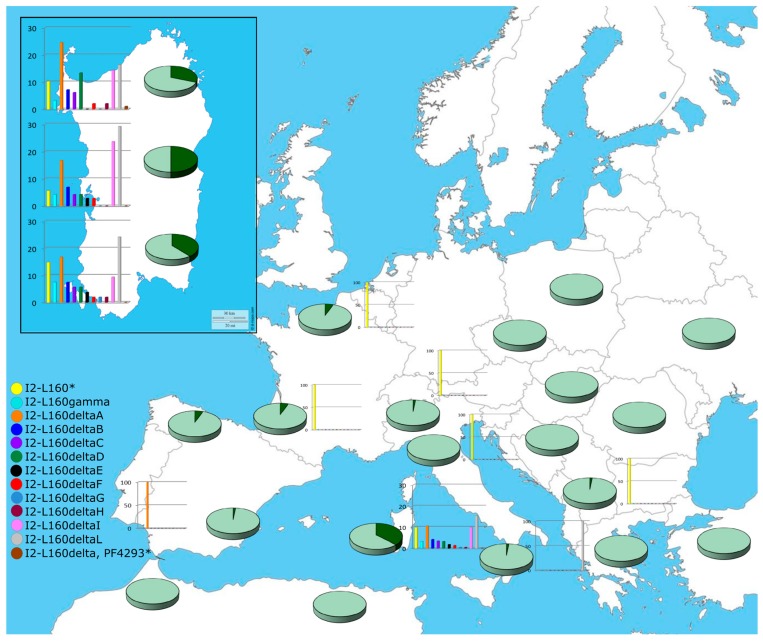
Geographical distribution of the Y-chromosome haplogroup I2-L160 and its sub-clades in Europe. Each pie represents a population and the dark green sector the percentage of I2-L160 (Tables S6 and S9); the histograms represent the percentages of the I2-L160 sub-clades according to the color codes shown on the figure. The inset shows the distribution of haplogroups and sub-haplogroups in the three geographical areas into which the Sardinian samples were subdivided according to the origin of their surnames.

On the whole, the Sardinian sample that we analyzed is characterized by a haplogroup heterogeneity of 0.8466 with high values in the northern (0.8814) and southern (0.8363) regions. The lowest heterogeneity in the central-eastern area (0.7469) reflects a reduction of the heterogeneity of all main haplogroups and can be explained by genetic drift due to long-term isolation in this region identified as the “archaic zone”. Indeed, it is reported that indigenous populations retreated to this mountainous region when Phoenicians and, later, Carthaginians colonized the southern part of the island [13]. The extraordinary low value of heterogeneity registered in the entire island for haplogroup I (HgI = 0.1898: 0.1766 in the North, 0.1264 in the Centre and 0.2857 in the South), which is almost completely represented by its sub-haplogroup I2-M26, which is rare elsewhere, can be explained only by a founder effect and genetic drift associated with the early peopling of Sardinia. In addition, taking into account that the three sub-clades stemming from the root of I2-M26 (I2-M26alfa (Z27361)-Table S6, I2-M26-beta-Z27401 and I2gamma-Z27138 [54]) are also present in other populations of Europe, haplogroup I-M26 must have been carried to Sardinia when it was already differentiated. Conversely, I2-M26delta, virtually only observed in Sardinia, likely originated and differentiated in situ on the island. Thus, the significantly higher frequency of some I2-M26delta (I2-L160 xZ27138) clades and the lack of chromosomes belonging to the rare clades I2-M26alpha (Z27361) and I2-M26beta (Z27401) in the “archaic zone” (Table S9) may reflect a second founder effect during the initial peopling of this area and/or genetic drift during the following long period of isolation (Figure 4).

For those haplogroups equally diversified in and outside Sardinia, it is difficult to discriminate between founder lineages and lineages that arrived later.

In brief, this study shows that the analysis of the Y-chromosome gene pool coupled with a sampling method based on the origin of the family name provides a greatly improved picture of the genetic structure of past population isolates. The application of this approach to a sample of Sardinian subjects carrying territorial monophyletic surnames (whose origin can be assigned to a specific location) allowed us to confirm the peculiarity of the Sardinian population in the European context and to detect ancient heterogeneities among the three main geographical areas of the island. We observed differences in the distribution not only of founding lineages and of lineages acquired through subsequent migrations, but also of sub-lineages that are Sardinian-specific. In particular, the high homogeneity displayed by the central-eastern sample is in agreement with the long-lasting isolation and founder and genetic drift effects of the “archaic zone”. In addition, the comparison of the haplotype distribution in past isolates with that displayed by recent data on ancient DNA from different geographical areas and archaeological periods offered the unique opportunity to better understand the genetic history and demography of this peculiar insular population.

## 4. Materials and Methods

### 4.1. The Sample

The sample consisted of 603 Sardinian males carrying Sardinian territorial monophyletic surnames [19] whose place of origin could be referenced to a specific Sardinian linguistic area (Figure S1) according to linguistic analyses [77,78].

These subjects were selected from a larger collection (*n* > 1000) of apparently unrelated healthy males, whose three-generation Sardinian origin was ascertained by interview, gathered thanks to the collaboration of many laboratories on the Sardinian territory, during different campaigns over more than 20 years. The selected samples were then assigned according to the place of origin of their surnames to the three large macro-areas of the island (North, Central-East and South-West Sardinia).

The “surname sampling approach”, previously applied in several studies [79,80,81,82], has been proved to be able to detect information earlier than the beginning of surnames history, which for Italy goes back to the Middle Age [19]. Indeed, in most societies, surnames are transmitted from father to child (just like Y chromosomes) and, being a linguistic attribution, reflect the identity and living area of the people. Thus, individuals bearing “monophyletic” surnames derived from words of the same local dialect likely share the same place of origin. In the case of Sardinia, the use of a sampling method based on the origin of monophyletic surnames, compared with the standard method based on the birthplace of the subjects, suggested a significant heterogeneity in the distribution of the Y-chromosome haplogroups [19].

### 4.2. DNA Analysis

Y-chromosome haplogroups were defined by hierarchical order analysis of 65 biallelic markers of the Male-Specific region of Y chromosome (MSY) (Figure 1), following the latest Y-chromosome phylogeny [54,83,84] and according to Battaglia et al. [71], Grugni et al. [3] and Karachanak et al. [85]. Seven markers, not included in previous papers, were analyzed as follows: L160 [86], M13 [87], M153 [44] and Z209 [86] using Restriction Fragment Length Polymorphism (RFLP) analysis, L2 [86] and V65 [73] using Deneturing High Performance Liquid Chromatography (DHPLC) and M282 [84] by sequencing.

All samples were also analyzed at ten STRs loci (DYS19, DYS388, DYS389I, DYS389B, DYS390, DYS391, DYS392, DYS393, YCAIIa and YCAIIb) using two multiplex reactions and a 3730 Applied Biosystems sequencer, as previously described [71]. Nomenclature details are available at the STRBase web site [88].

### 4.3. Statistical Analysis

Considering the results previously obtained [19], three main areas of Sardinia were considered (Figure S1): North (linguistic zones 1–5 and 23), Central-East (linguistic zones 6–9, 11, 14 and 15) and South-West (linguistic zones 10, 12, 13 and 16–22).

Haplogroup distributions were compared through the Chi Square Test of independence using the Xlstat add-on for Excel. Haplogroup heterogeneity (H) was computed using Nei’s [89] standard method. PC analysis was performed on Y-chromosome haplogroup frequencies disregarding those lower than 5% through prcomp() function on R (R Core Team 2017) and setting as TRUE the center and scale function.

MDS was performed using the Xlstat add-on for Excel and R_ST_ values [90] calculated on STRs haplotypes associated to haplogroups G2-L91 and I2-M26(xL160). Genetic structure was examined using analysis of molecular variance (AMOVA, [91]) using Arlequin software Ver 3.5 (Laurent Excoffier and Heidi Lischer, Bern, Switzerland).

Within specific haplogroups, median-joining (MJ) networks [92] were constructed using Network 4.6.0.0 [93], after processing the data with the reduced-median method [94] and weighting the STR loci proportionally to the inverse of the repeat variance. Time estimates were calculated only when more than five observations per population/region were available. Coalescent times were defined using the methodology of Zhivotovsky et al. [95] as modified according to Sengupta et al. [96] by using a microsatellite evolutionary effective mutation rate of 6.9 × 10^−4^ per generation (25 years).

## Supplementary Materials

Supplementary materials can be found at https://www.mdpi.com/1422-0067/20/22/5763/s1.

## Abbreviations

BCE	Before common era
CE	Common era
DHPLC	Denaturating high performance liquid chromatography
ky	Kilo years
kya	Kilo years ago
MDS	Multi-dimensional scaling analysis
MSY	Male specific region of the Y chromosome
PCA	Principal component analysis
RFLP	Restriction fragment length polymorphism
STR	Short tandem repeat
