# Supplementary material for: Y-chromosome and Surname Analyses for Reconstructing Past Population Structures: The Sardinian Population as a Test Case
Source: Int J Mol Sci. 2019 Nov 16;20(22):5763. doi: 10.3390/ijms20225763 (PMC6888588; doi:10.3390/ijms20225763)
Supplement: Supplementary file 1 [file ijms-20-05763-s001.zip › Figure S1.pdf]

Figure S1. Linguistic areas of Sardinia.

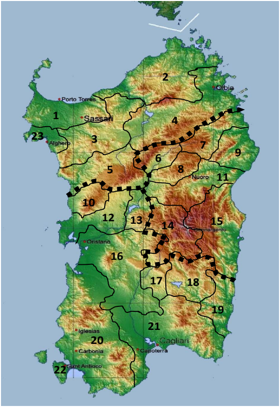

NORTHER AREA:
- 1 Sassarese
- 2 Gallurese
- 3 North-West Logudoro
- 4 East Logudoro
- 5 South Logudoro – Planargia
- 23 Alghero

CENTRAL-EASTERN AREA:
- 6 Goceano
- 7 Circondario di Bitti
- 8 Nuorese
- 9 Baronia di Orosei Siniscola
- 11 Fonni - Barbagia di Ollolai
- 14 Barbagia di Belvì
- 15 Ogliastra

SOUTH-WESTERN AREA:
- 10 Montiferru
- 12 North Campidano of Oristano
- 13 Media valle del Tirso
- 16 South Campidano of Oristano Arborea – Trexenta
- 17 Trexenta – Parteolla
- 18 Transition between Gerrei and Trexenta
- 19 Sarrabus
- 20 Sulcis-Iglesiente
- 21 Campidano di Cagliari
- 22 Carloforte

Dashed lines indicate the three main linguistic areas of Sardinia.
