# Supplementary material for: Y-chromosome and Surname Analyses for Reconstructing Past Population Structures: The Sardinian Population as a Test Case
Source: Int J Mol Sci. 2019 Nov 16;20(22):5763. doi: 10.3390/ijms20225763 (PMC6888588; doi:10.3390/ijms20225763)
Supplement: Supplementary file 1 [file ijms-20-05763-s001.zip › Figure S2.pdf]

Figure S2. Network of six-loci Y-STR haplotypes (DYS389I, DYS389B, DYS390, DYS391, DYS392 and DYS393) belonging to haplogroup G2-L91.

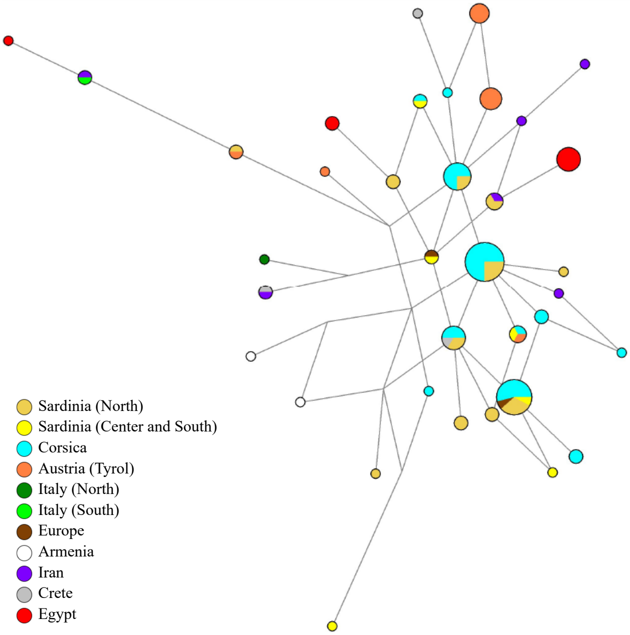

Network analysis was performed on Sardinian samples in comparison with all the samples available in the literature analyzed for the same set of STRs (Table S4). Circles and colored sectors are proportional to the number of subjects, with the smallest circle equal to one Y chromosome; connecting lines are proportional to the number of mutations.
