# Supplementary material for: Y-chromosome and Surname Analyses for Reconstructing Past Population Structures: The Sardinian Population as a Test Case
Source: Int J Mol Sci. 2019 Nov 16;20(22):5763. doi: 10.3390/ijms20225763 (PMC6888588; doi:10.3390/ijms20225763)

Figure S3. Two-dimensional plot obtained from MDS analysis based on pairwise RST values for six-loci Y-STR haplotypes (DYS389I, DYS389B, DYS390, DYS391, DYS392 and DYS393) associated to haplogroup G2-L91 observed in Sardinians and other populations of the Mediterranean Basin.

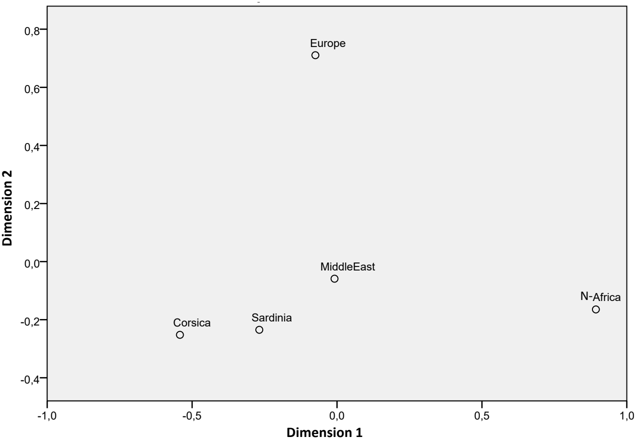

Supplement: Supplementary file 1 [file ijms-20-05763-s001.zip › Figure S3.pdf]
