# Supplementary material for: Y-chromosome and Surname Analyses for Reconstructing Past Population Structures: The Sardinian Population as a Test Case
Source: Int J Mol Sci. 2019 Nov 16;20(22):5763. doi: 10.3390/ijms20225763 (PMC6888588; doi:10.3390/ijms20225763)
Supplement: Supplementary file 1 [file ijms-20-05763-s001.zip › Figure S4.pdf]

Figure S4. Network of Y-STR haplotypes belonging to haplogroup I2-L160.

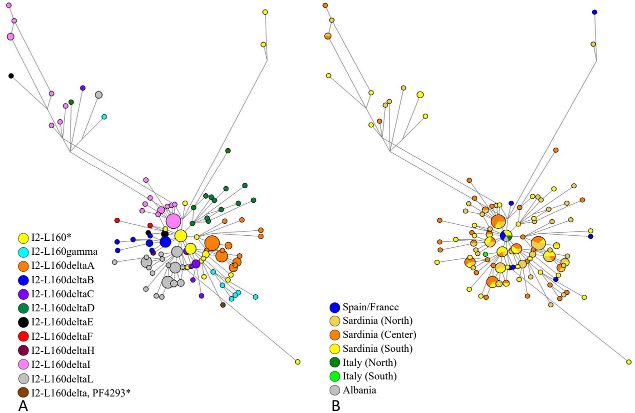

Network analysis was performed on Sardinian samples in comparison with all the samples available in the literature for this haplogroup (Table S7). Circles and colored sectors are proportional to the number of subjects, with the smallest circle equal to one Y chromosome; connecting lines are proportional to the number of mutations.
