# Supplementary material for: Y-chromosome and Surname Analyses for Reconstructing Past Population Structures: The Sardinian Population as a Test Case
Source: Int J Mol Sci. 2019 Nov 16;20(22):5763. doi: 10.3390/ijms20225763 (PMC6888588; doi:10.3390/ijms20225763)
Supplement: Supplementary file 1 [file ijms-20-05763-s001.zip › Figure S5.pdf]

Figure S5. Network of Y-STR haplotypes belonging to I2-M26(xL160) Y chromosomes.

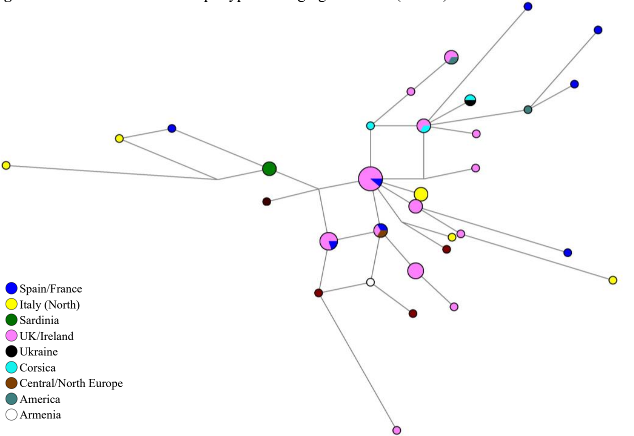

Network analysis was performed on Sardinian samples in comparison with all the I2-M26(xL160) samples available in the literature (Table S8). Circles and colored sectors are proportional to the number of subjects, with the smallest circle equal to one Y chromosome; connecting lines are proportional to the number of mutations.
