# Supplementary material for: Y-chromosome and Surname Analyses for Reconstructing Past Population Structures: The Sardinian Population as a Test Case
Source: Int J Mol Sci. 2019 Nov 16;20(22):5763. doi: 10.3390/ijms20225763 (PMC6888588; doi:10.3390/ijms20225763)
Supplement: Supplementary file 1 [file ijms-20-05763-s001.zip › Figure S7.pdf]

Figure S7. Y-chromosome haplogroup distribution per macro-area of ancient Sardinian samples. For each bar plot: the name of haplogroups on the left side, the temporal range of the ancient samples, on the right side.

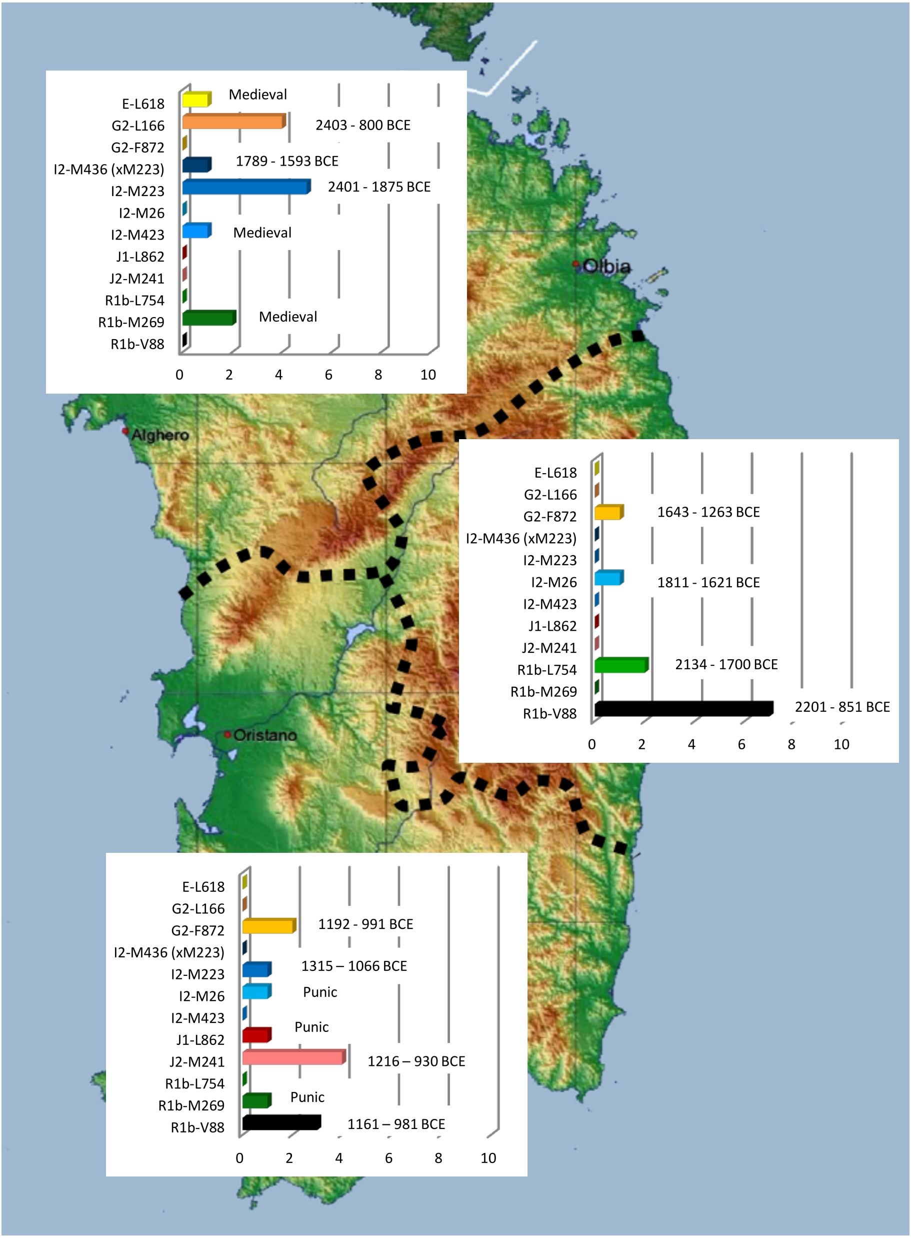

Data from Marcus et al. and Fernandes et al.
